# Supplementary material for: Monkeys mutant for PKD1 recapitulate human autosomal dominant polycystic kidney disease
Source: Nat Commun. 2019 Dec 11;10:5517. doi: 10.1038/s41467-019-13398-6 (PMC6904451; doi:10.1038/s41467-019-13398-6)
Supplement: Supplementary file 1 — Supplementary Information [file 41467_2019_13398_MOESM1_ESM.pdf]

Monkeys mutant for *PKD1* recapitulate human autosomal dominant polycystic kidney disease.

Tsukiyama, Kobayashi and Nakaya et al.

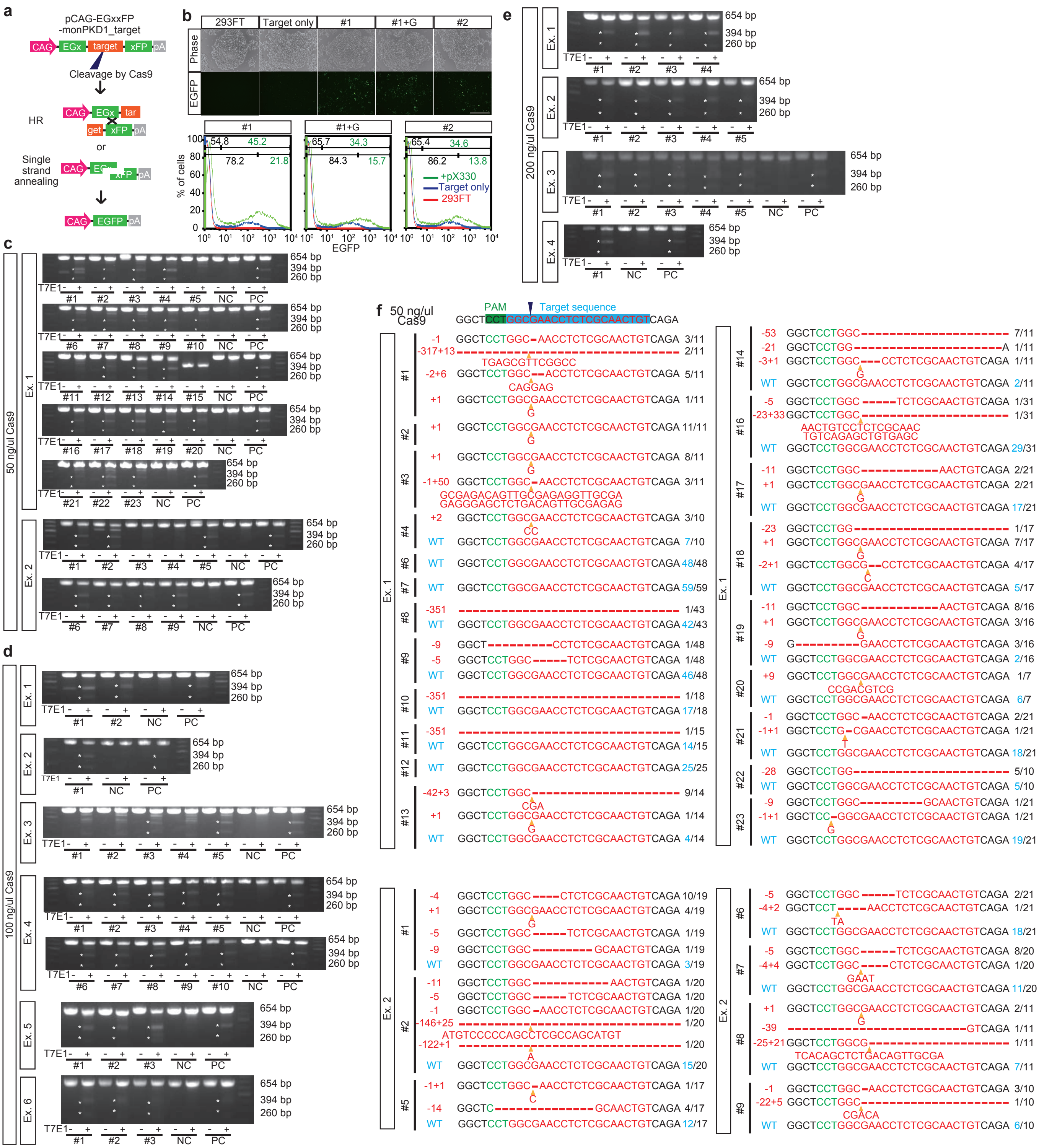

Supplementary Fig. 1. Single-strand annealing (SSA) assay and embryo genotypes. (a) Schematic diagram of the SSA assay using pCAG-EGxxFP. “HR” indicates homologous recombination. (b) EGFP signals 2 days after transfection of an EGxxFP vector and CRISPR/Cas9 vectors into 293FT cells. (c-e) T7E1 assay of embryos injected with 50, 100, or 200 ng/µl Cas9 mRNA and 50 ng/µl sgRNA. Asterisks indicate positive bands. “NC” indicates negative control, in which wild-type DNAs were used as the PCR template. “PC” indicates positive control, in which mutated DNAs were used as the PCR template. (f) Sequences of sgRNA targets in each mRNA-injected embryo. Source data are provided as a Source Data file.

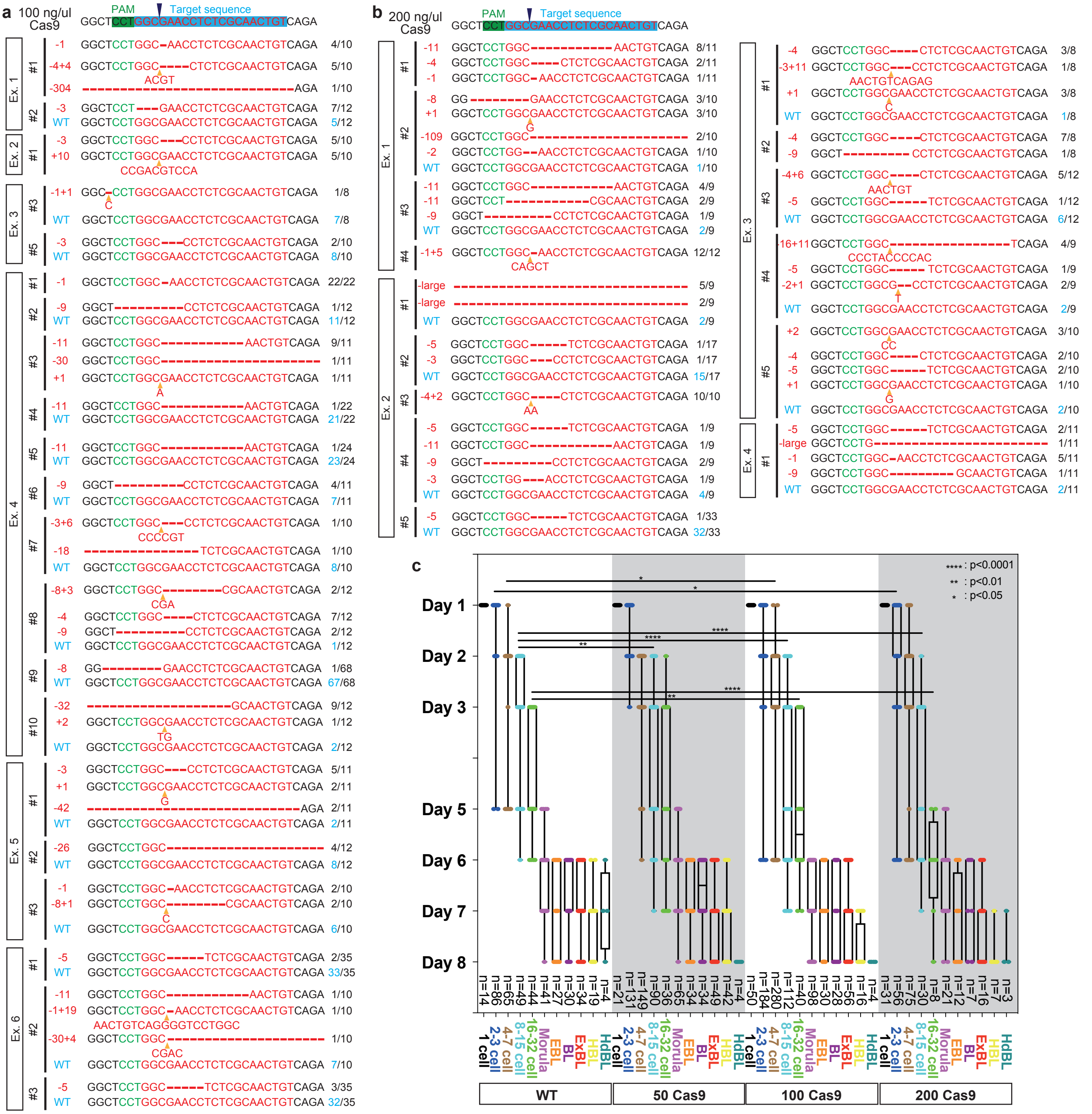

Supplementary Fig. 2. Genotypes and development of embryos. (a and b) Sequences of sgRNA targets in each mRNA-injected embryo. (c) Plot of days at each developmental stage for each type of mRNA-injected embryo. Source data are provided as a Source Data file.

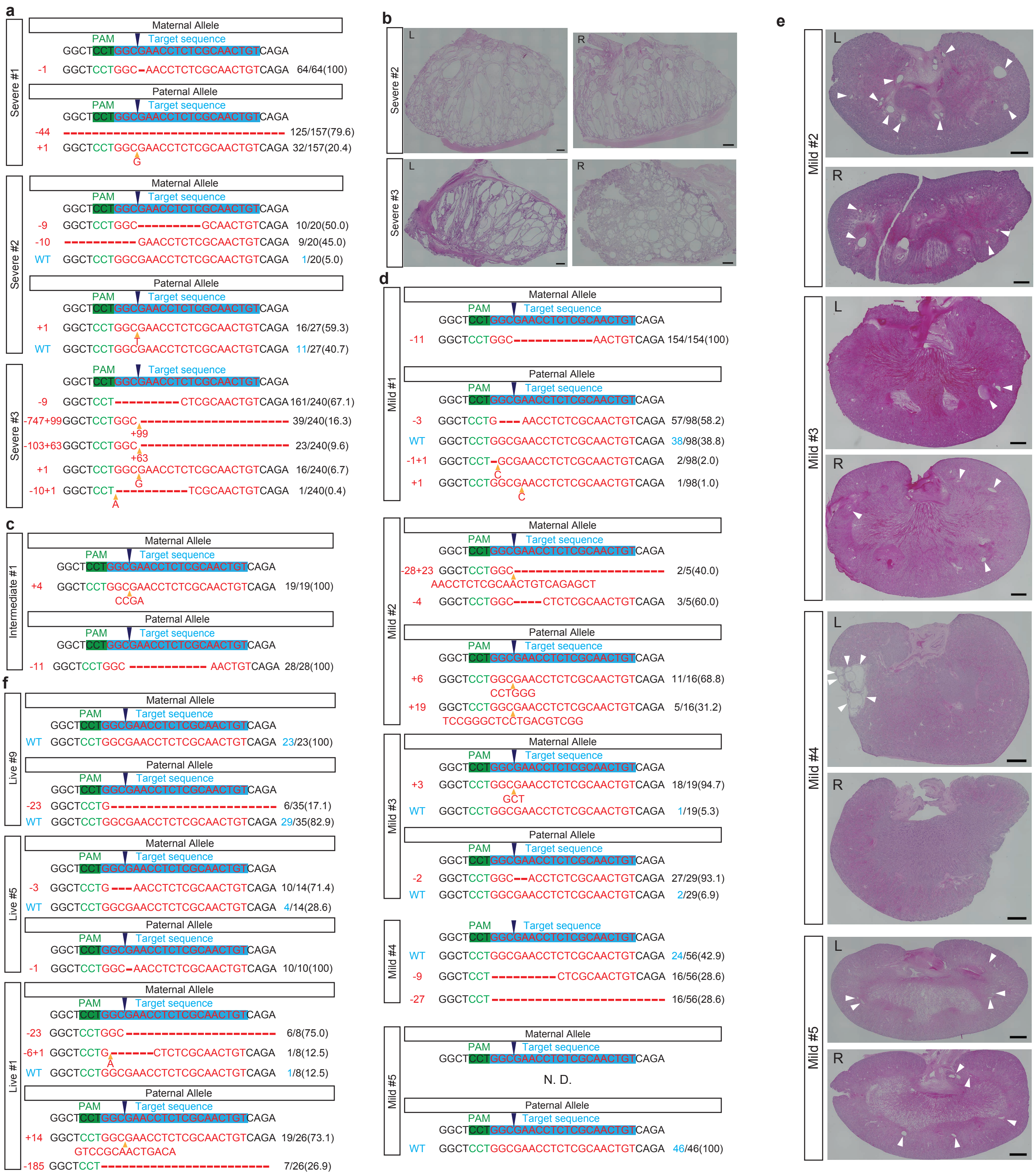

Supplementary Fig. 3. Genotypes and H&E-stained images of monkeys. (a) Sequences of sgRNA targets in the genomes of monkeys with severe-type kidneys. (b) Low-power, H&E-stained images of severe-type kidneys. “L” indicates left kidneys, and “R” indicates right kidneys. Scale bar, 1 mm. (c) Sequences of sgRNA targets in the genome of a monkey with intermediate-type kidneys. (d) Sequences of sgRNA targets in the genomes of monkeys with mild-type kidneys. N.D. indicates not detected. (e) Low-power, H&E-stained images of mild-type kidneys. Arrowheads indicate cyst formation. Scale bar, 1 mm. (f) Sequences of sgRNA targets in the genomes of PKD1-mutated live monkeys.

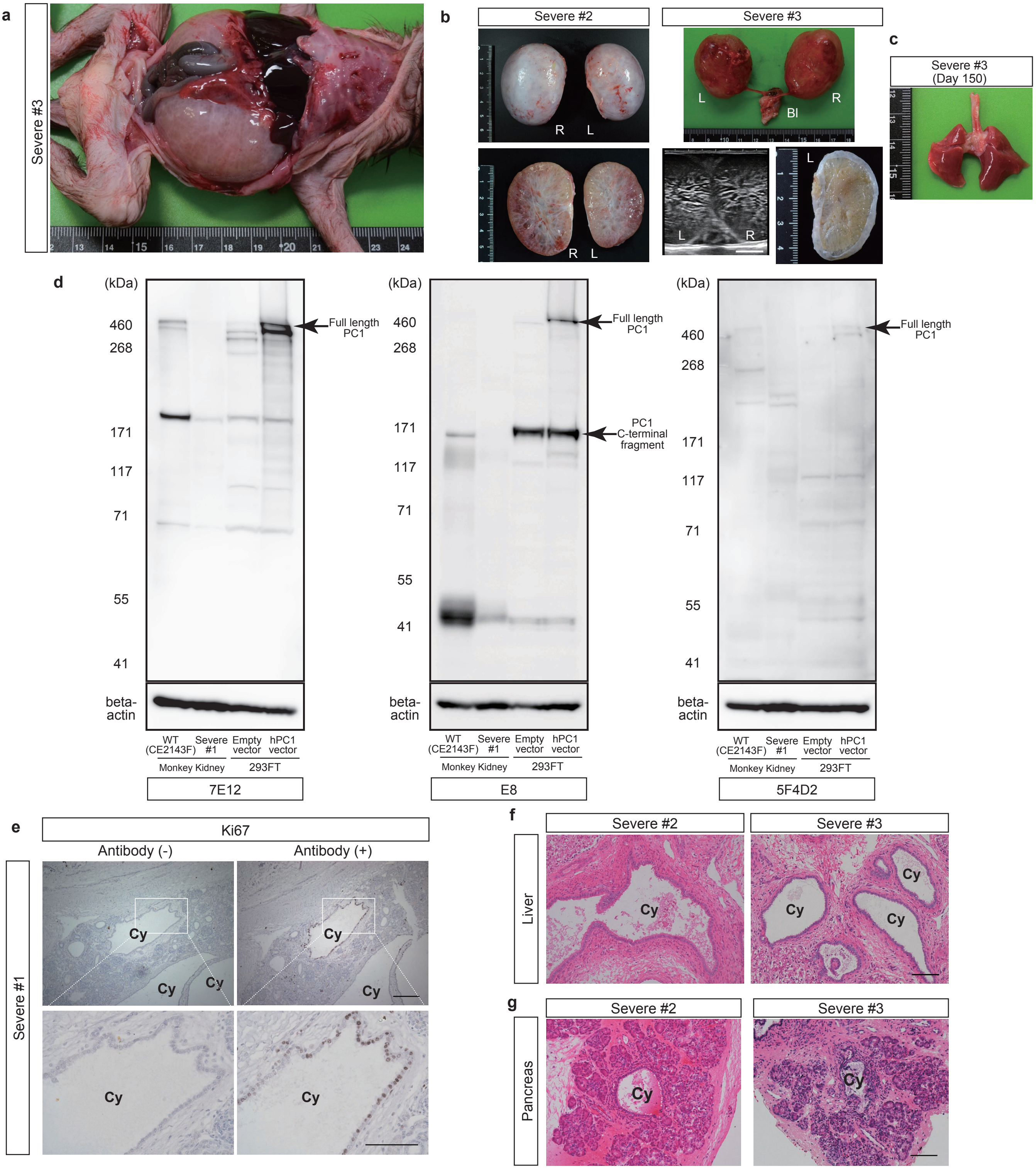

Supplementary Fig. 4. Severe phenotypes and expression of nephron segment markers in monkeys with severe-type kidneys. (a) A monkey with enlarged severe-type kidneys. (b) Ultrasonographic and cross-section appearance of severe-type kidneys. “L” indicates left kidneys, and “R” indicates right kidneys. “Bl” indicates a bladder. Scale bar in the ultrasonography image, 10 mm. (c) The immature lungs of a monkey with severe-type kidneys. “Day” indicates the day of abortion. (d) The expression levels of PC1 protein. “Empty vector” and “hPC1 vector” indicate the 293FT samples transfected with empty or human PC1-overexpression vectors, respectively. “7E12,” “E8,” and “5F4D2” indicate the clone names of anti-PC1 monoclonal antibodies. (e) Expression of Ki67 in cystic cells in a severe-type kidney. Small boxes indicate the regions that show at high magnification in the low column. “Cy” indicates cysts. Scale bar, 100 µm. (f) Liver cysts. “Cy” indicates cysts. Scale bar, 100 µm. (g) Pancreatic cysts. “Cy” indicates cysts. Scale bar, 100 µm. Source data are provided as a Source Data file.

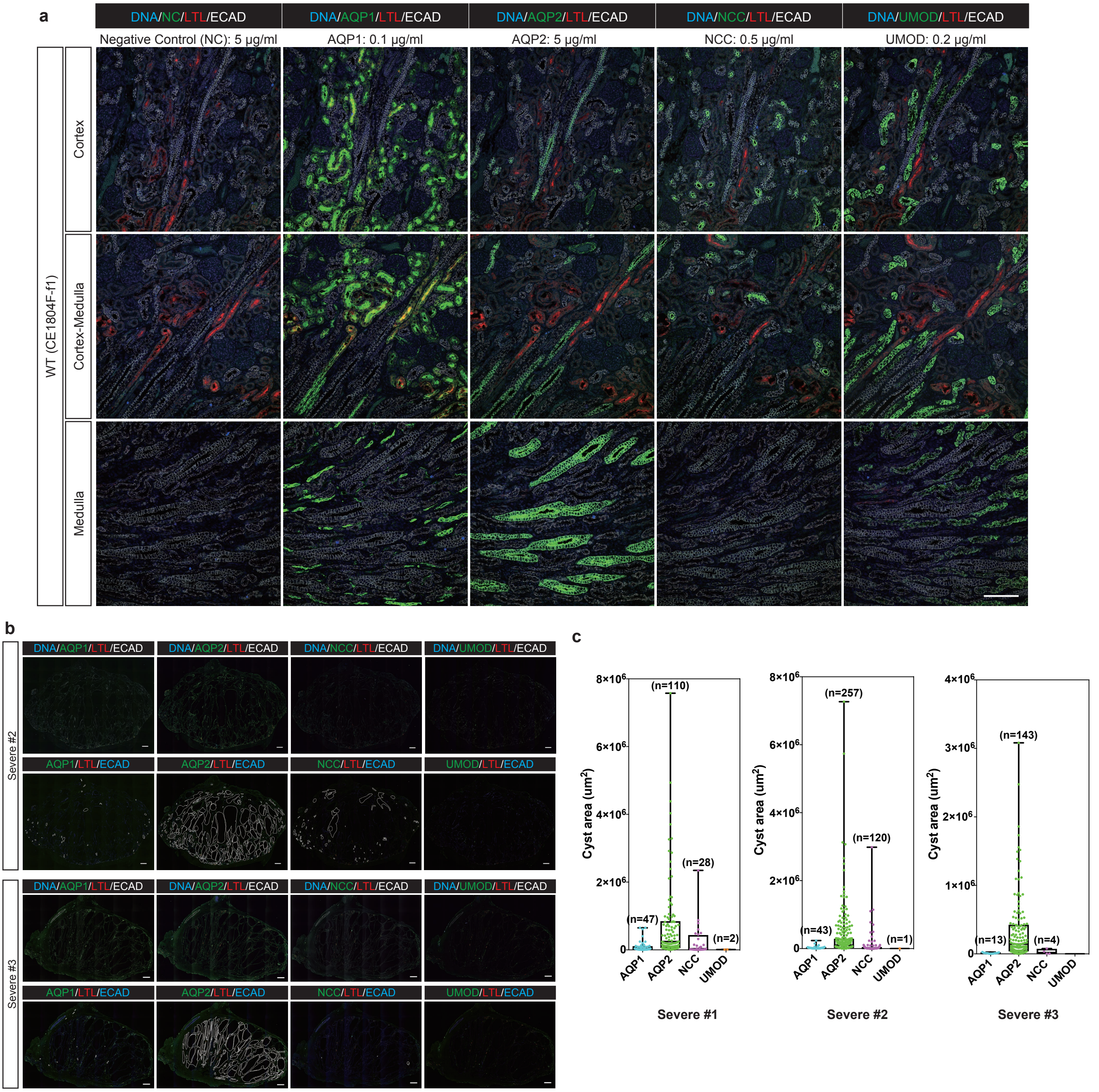

Supplementary Fig. 5. Expression of nephron segment markers in monkeys with severe-type kidneys. (a) Examination for autofluorescence or non-specific staining using negative control immunoglobulin. Scale bar, 100 µm. (b) Expression of nephron segment markers in severe-type kidneys. Cystic areas that are positive for AQP1, AQP2, NCC, or UMOD are surrounded by white lines. Scale bar, 1 mm. (c) Box plots of the areas of AQP1-, AQP2-, NCC-, or UMOD-positive cysts in severe-type kidneys. The top and bottom edges of boxes indicate the first and third quartiles, respectively; the center lines indicate the medians; and the ends of whiskers indicate the maximum and minimum values, respectively. Source data are provided as a Source Data file.

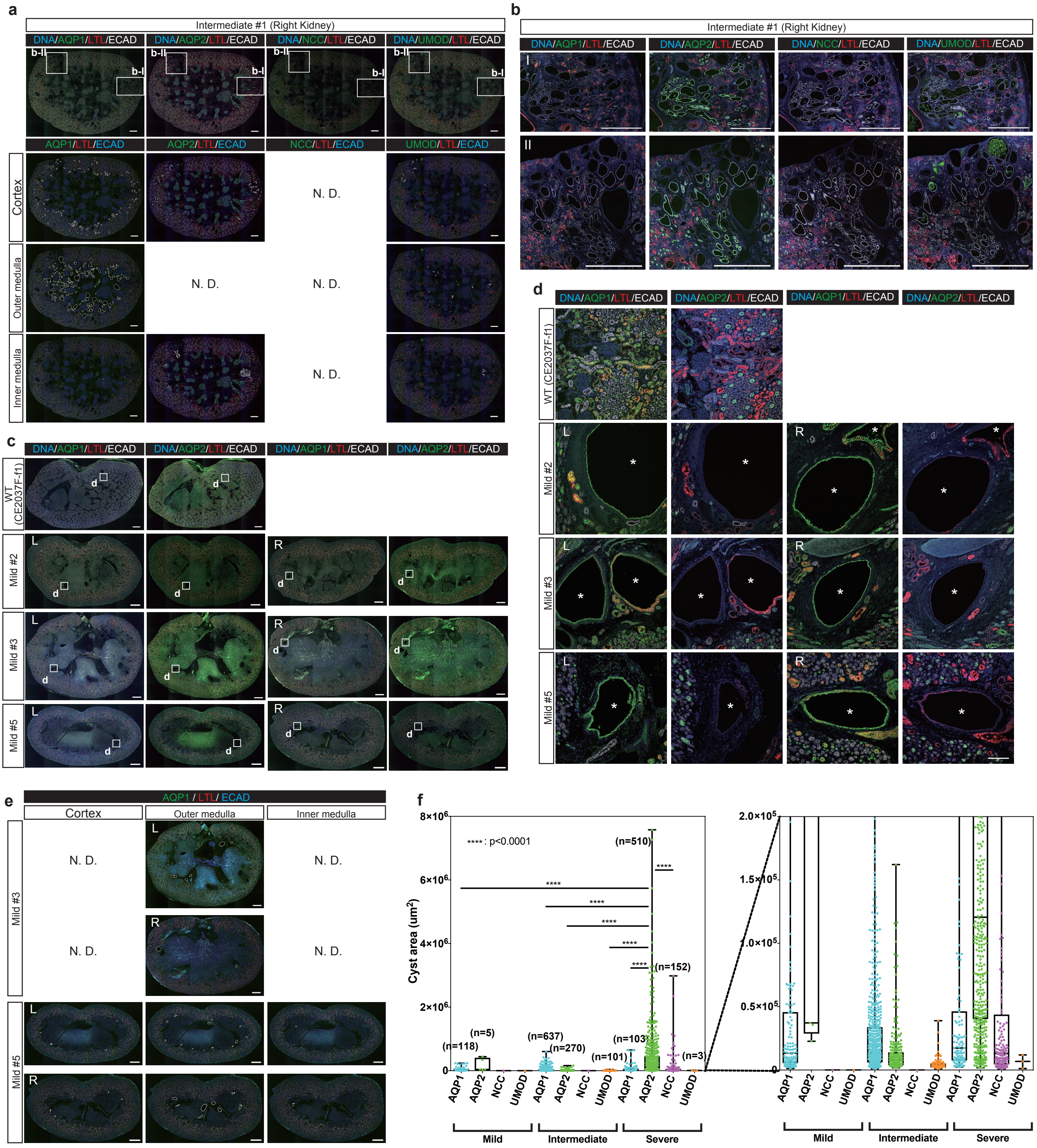

Supplementary Fig. 6. Expression of nephron segment markers in monkeys with intermediate- and mild-type kidneys. (a) Expression of nephron segment markers in an intermediate kidney. Small boxes indicate the regions shown in high magnification in (b). Cystic areas that are positive for AQP1, AQP2, or UMOD in the cortex, outer medulla, or inner medulla are surrounded by white lines. Scale bar, 1 mm. (b) Expressions of nephron segment markers in an intermediate-type kidney compartment containing multiple cysts similar to those in severe-type kidneys. Scale bar, 1 mm. (c) Expression of nephron segment markers in mild-type kidneys. Small boxes indicate the regions shown in high magnification in (d). “L” indicates left kidneys, and “R” indicates right kidneys. Scale bar, 1 mm. (d) Representative AQP1-positive and AQP2-negative cysts in mild-type kidneys. Asterisks indicate cysts. Scale bar, 100 μm. (e) Expression of nephron segment markers in mild-type kidneys. Cystic areas that are positive for AQP1 in the cortex, outer medulla, or inner medulla, are surrounded by white lines. Scale bar, 1 mm. (f) Comparison of cystic areas among mild-, intermediate-, and severe-type kidneys. The top and bottom edges of boxes indicate the first and third quartiles, respectively; the center lines indicate the medians; and the ends of whiskers indicate the maximum and minimum values, respectively. Source data are provided as a Source Data file.

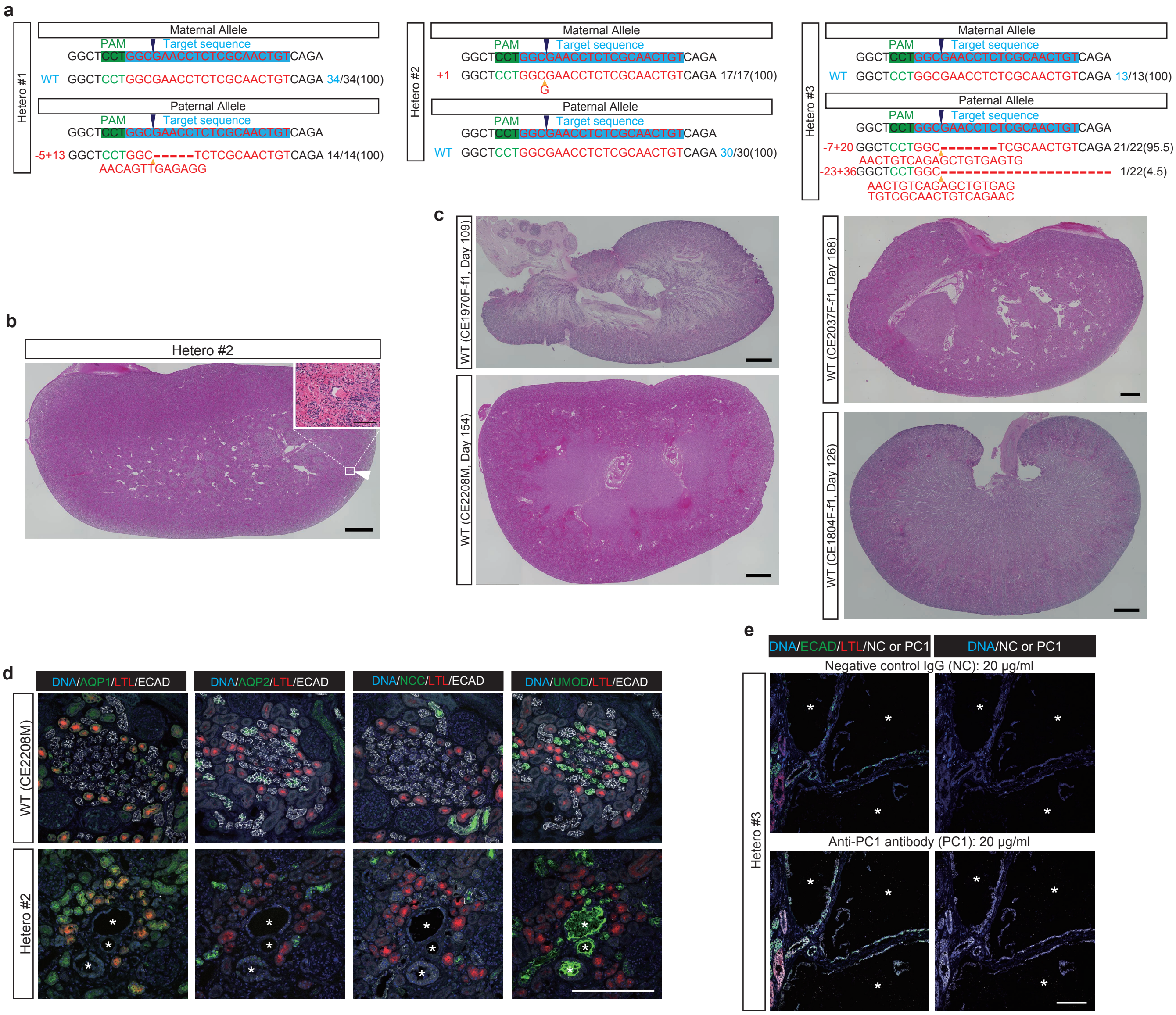

Supplementary Fig. 7. Genotypes and phenotypes of exon 2 heterozygous monkeys. (a) Sequences of sgRNA targets in the genomes of exon 2 heterozygous monkeys. (b) Low-power, H&E-staining images of a heterozygous kidney. Arrowheads indicate cyst formation. Scale bar in a large image, 1 mm. Scale bar in a small box, 100 μm. (c) H&E-stained images of kidneys of wild-type monkeys. Low-power, H&E-stained images of the kidneys. Days indicate the aborted days. Scale bar, 1 mm. (d) Representative ECAD-positive cysts in a heterozygous kidney. Asterisks indicate cysts. Scale bar, 100 μm. (e) Expressions of PC1 in heterozygous kidneys. Representative PC1-positive cysts in Hetero #3 are shown. Asterisks indicate cysts. Scale bar, 100 μm.

a

Recipients	Methods	gRNA types	Concentrations	Mutation detection rates (%)		Mosaic (%)	ET
				Maternal	Paternal		
Pronuclear Zygote	Cytoplasmic Injection	mRNA	200 ng/ul	0 / 90 (0)	35 / 35 (100)	3 / 8 (37.5)	
MII Oocyte	Co-injection with sperm	RNP complex	200 ng/ul	1 / 104 (0.96)	72 / 72 (100)	2 / 16 (12.5)	35
			20 ng/ul	0 / 26 (0)	6 / 23 (26.1)	4 / 5 (80.0)	
ICSI Embryo	Electroporation	RNP complex	200 ng/ul	0 / 24 (0)	29 / 29 (100)	0 / 5 (0)	23

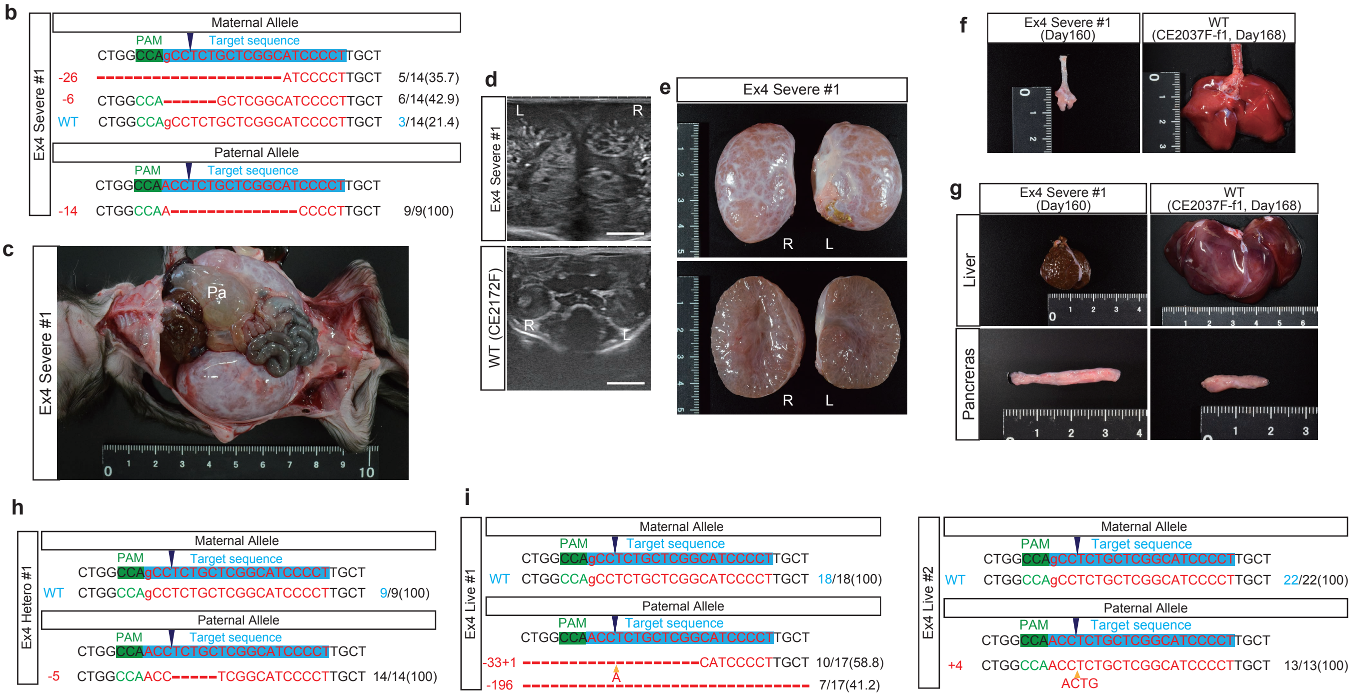

Supplementary Fig. 8. Genotypes and phenotypes of exon 4 mutated monkeys. (a) The genotyping results of exon 4–mutated blastocyst embryos. The mutation detection rate per sequenced DNA and mosaic rate per embryo are shown. “ET” indicates the number of transferred embryos. (b) Sequences of gRNA targets in the genome of an exon 4 mutated monkey, Ex4 Severe #1. (c) Enlarged kidneys and pancreas in monkey Ex4 Severe #1. “Pa” indicates the enlarged pancreas. (d) Ultrasonographic appearance of the kidneys. “L” indicates left kidneys, and “R” indicates right kidneys. Scale bar, 10 mm. (e) The cross-sectional appearance of the kidneys. (f) Immature lung of monkey Ex4 Severe #1. “Day” indicates day of abortion. (g) Gross appearance of the liver and pancreas of monkey Ex4 Severe #1. “Day” indicates day of abortion. (h) Sequences of gRNA targets in the genome of an exon 4 mutated aborted monkey, Ex4 Hetero #1. (i) The sequences of gRNA target in the genomes of exon 4 mutated live monkeys, Ex4 Live #1 and #2.

									Maternal genotype (%)				Paternal genotype (%)				Allele-indistinguishable genotype (%)				Cyst formation		
ID	Institute ID	Sex	Survival	Day of abortion	Day of birth	Day of death	Sequenced	Mosaicism	Frameshift	In-frame (≧3AA)	In-frame (≦2AA) or missense	WT	Frameshift	In-frame (≧3AA)	In-frame (≦2AA) or missense	WT	Frameshift	In-frame (≧3AA)	In-frame (≦2AA) or missense	WT	Kidney	Liver	Pancreas
**Severe #1**	**CE2104F-f1**	Female	Dead	144			221	Mosaic	100				100								**+++**	**+**	**+**
**Severe #2**	**CE2154F-f1**	Male	Dead	163			47	Mosaic	45	50		5	59.3			40.7					**+++**	**+**	**+**
**Severe #3**	**CE1950F-f1**	Female	Dead	150			240	Mosaic									32.5	67.5			**+++**	**+**	**+**
**Intermediate #1**	**CE2170M**	Male	Dead		161	2	47	Non-mosaic	100				100								**++**	**-**	**-**
**Mild #1**	**CE1788F-f1**	Female	Dead	153			252	Mosaic	100				0		60.2	38.8					**+**	**-**	**-**
**Mild #2**	**CE2015F-f1**	Female	Dead	114			21	Mosaic	100				31.2		68.8						**+**	**-**	**-**
**Mild #3**	**CE1696F-f2**	Male	Dead	148			48	Mosaic			94.7	5.3	93.1			6.9					**+**	**ND**	**ND**
**Mild #4**	**CE2158F**	Female	Dead		160	8	32	Mosaic										28.6	28.6	42.8	**+**	**-**	**-**
**Mild #5**	**CE1788F-f2**	Female	Dead	125			46	ND													**+**	**ND**	**ND**
**Hetero #1**	**CE2002F-f1**	Female	Dead	128			48	Non-mosaic				100	100								**+**	**-**	**-**
**Hetero #2**	**CE2196F**	Female	Dead		160	4	47	Non-mosaic	100							100					**+**	**ND**	**ND**
**Hetero #3**	**CE2214F**	Female	Dead		153	216	54	Mosaic				100	100								**+**	**ND**	**ND**
**Live #1**	**CE2215M**	Male	Live		163		34	Mosaic	87.5			12.5	100								**++**	**ND**	**ND**
**Live #2**	**CE2209F**	Female	Live		156		33	Mosaic	100				28.6	66.7		4.7					**+**	**ND**	**ND**
**Live #3**	**CE2216F**	Female	Live		164		18	Mosaic	50	20		30	75			25					**+**	**ND**	**ND**
**Live #4**	**CE2225M**	Male	Live		164		35	Mosaic	100				39.1			60.9					**++**	**ND**	**ND**
**Live #5**	**CE1995F**	Female	Live		160		24	Mosaic			71.4	28.6	100								**+**	**ND**	**ND**
**Live #6**	**CE2190F**	Female	Live		149		30	Mosaic	69.2			30.8	35.3			64.7					**+**	**ND**	**ND**
**Live #7**	**CE2191F**	Female	Live		165		34	Mosaic		18.2		81.8	69.6	30.4							**+**	**ND**	**ND**
**Live #8**	**CE2197F**	Female	Live		167		35	Mosaic	8.7	4.3		87	75	25							**+**	**ND**	**ND**
**Live #9**	**CE1987M**	Male	Live		156		58	Mosaic				100	17.1			82.9					**-**	**ND**	**ND**
**Live #10**	**CE2189F**	Female	Live		162		34	Mosaic	36.4			63.6				100					**-**	**ND**	**ND**
**No kidney sample (Mosaic) #1**	**CE1980F-f1**	Female	Dead	78			53	Mosaic									96.2			3.8	**ND**	**ND**	**ND**
**No kidney sample (Mosaic) #2**	**CE2102F-f1**	ND	Dead	175			23	Mosaic				100	92.9			7.1					**++**	**ND**	**ND**
**No kidney sample (Mosaic) #3**	**CE1942F-f1**	Female	Dead	141			36	Mosaic	33.3			66.7				100					**ND**	**ND**	**ND**
**No kidney sample (Mosaic) #4**	**CE2110F-f1**	ND	Dead	165			24	Mosaic				100			5	95					**ND**	**ND**	**ND**
**No kidney sample (Hetero) #1**	**CE1665F-f3**	ND	Dead	51			22	Non-mosaic	100							100					**ND**	**ND**	**ND**
**No kidney sample (Hetero) #2**	**CE2075F-f1**	ND	Dead	103			24	Mosaic				100	100								**ND**	**ND**	**ND**
**No kidney sample (Genotype ND) #1**	**CE2016F-f1**	ND	Dead	83				ND													**ND**	**ND**	**ND**
**Ex4 Severe #1**	**CE2344M**	Male	Dead	160			23	Mosaic	35.7		42.9	21.4	100								**+++**	**+**	**+**
**Ex4 Severe #2**	**CE2348M**	Male	Dead		158	5	24	Mosaic	30.8			69.2	100								**+++**	**ND**	**ND**
**Ex4 Hetero #1**	**CE0362F-f1**	Male	Dead	159			23	Non-mosaic				100	100								**+**	**ND**	**ND**
**Ex4 Live #1**	**CE2345F**	Female	Live		161		35	Mosaic				100	100								**+**	**ND**	**ND**
**Ex4 Live #2**	**CE2346F**	Female	Live		157		35	Non-mosaic				100	100								**+**	**ND**	**ND**
**Ex4 Live #3**	**CE2347M**	Male	Live		152		34	Non-mosaic				100			100						**-**	**ND**	**ND**
**Ex4 Live #4**	**CE2373F**	Female	Live		150		36	Non-mosaic				100	100								**+**	**ND**	**ND**
**Ex4 Live #5**	**CE2374F**	Female	Live		146		36	Non-mosaic				100	100								**ND**	**ND**	**ND**
**Ex4 No kidney sample (Hetero) #1**	**CE2126F-f2**	ND	Dead	63			36	Non-mosaic				100	100								**ND**	**ND**	**ND**
**Ex4 No kidney sample (Hetero) #2**	**CE2025F-f1**	Male	Dead	140			23	Non-mosaic				100	100								**ND**	**ND**	**ND**
**Ex4 No kidney sample (Hetero) #3**	**CE2143F-f1**	Female	Dead	148			23	Non-mosaic				100	100								**ND**	**ND**	**ND**
**Ex4 No kidney sample (Hetero) #4**	**CE2141F-f2**	Female	Dead	154			24	Non-mosaic				100	100								**ND**	**ND**	**ND**

Supplementary Table 1. Fetuses and offspring with their genotypes and pathologies. “Sequenced” indicates the number of sequences examined in this analysis. “+++,” “++,” “+,” and “–” indicate the severity of cyst formation. “ND” indicates that the cyst formation was not determined. Source data are provided as a Source Data file.

Supplementary Table 2. Oligonucleotides used in this study.

Names	Forward	Reverse	For
monPKD1_Ex2_1_#1_F,R	caccACAGTTGCGAGAGGTTCGCC	aaacGGCGAACCTCTCGCAACTGT	sgRNA cloning
monPKD1_Ex2_1_#1+G_F,R	caccGACAGTTGCGAGAGGTTCGCC	aaacGGCGAACCTCTCGCAACTGTC	sgRNA cloning
monPKD1_Ex2_1_#2_F,R	caccCAGCGCCCGGAGCAAGTTAT	aaacATAACTTGCTCCGGGCGCTG	sgRNA cloning
monPKD1_target_EcoRI_F, NheI_R	ataGAATTCgcgactgtggacaagaaattgcaggac	ataGCTAGCgccactgatacccacccaaagaaccac	SSA assay
T7-Cas9_F,R	ttaatacgactcactatagGGAGAATGGACTATAAGGACCACGAC	GCGAGCTCTAGGAATTCTTAC	In vitro transcription
T7-sgRNA_monPKD1_Ex2_1_1_F,R	ttaatacgactcactataggACAGTTGCGAGAGGTTCGCC	AAAAGCACCGACTCGGTGCC	In vitro transcription
monPKD1_target_EcoRI_F, NheI_R	ataGAATTCgcgactgtggacaagaaattgcaggac	ataGCTAGCgccactgatacccacccaaagaaccac	Genotyping (Ex2)
monPKD1_target_EcoRI_F, outer_R3	ataGAATTCgcgactgtggacaagaaattgcaggac	TGTCAATGGTCAGTGTGGGCCTAAGATG	Genotyping (Ex2 long)
monPKD1_Ex4_geno_F,R	TCCCATTCCAGGCTTGAGACCAGATC	TGTCAGGGAGGCAGGCGATATAC	Genotyping (Ex4)
monPKD1_Ex4_geno_F,Int4	TCCCATTCCAGGCTTGAGACCAGATC	ATAGCTAGCCAGGGAAGACATGCTGGAGGAGGGTTG	Genotyping (floxed

_target_NheI_R			Ex4, RFLP assay)
monPKD1_target_EcoRI_F,Int4_target_NheI_R	ataGAATTCgcgactgtggacaagaaattgcaggac	ATAGCTAGCCAGGGAAGACATGCTGGAGGAGGGTTG	Genotyping (floxed Ex4, Sequencing)
monPKD1_flox_Ex4_5_arm_F, BbsI_R	CCTCTCTTCCAGGGATATAAGCAACAACAAG	GAAGACAATTATCCGGCAACCAGGCCCTGGAG	Long ssODN production (1st PCR)
monPKD1_flox_Ex4_BbsI_loxP_F, R	GAAGACAAATAACTTCGTATAGCATACATTATACGAAGTTATTAAGGGGCTGGTGTAGACCCTTCCCAC	GAAGACAAATAACTTCGTATAATGTATGCTATACGAAGTTATCTGCTCTCTTGGCCCGGAGGC	Long ssODN production (1st PCR)
monPKD1_flox_Ex4_3_arm_BbsI_F, R	GAAGACAATTATCATGGGAGCCTGTGAGTGTGGC	GAACAGAAGGACAGGCAGGCGAAG	Long ssODN production (1st PCR)
monPKD1_flox_Ex4_5_BamHI_BbsI_F, R	CCTCTCTTCCAGGGATATAAGCAACAACAAG	GAAGACAAATCCCGAGCAGAGGCTGGCCAGC	Long ssODN production (2nd PCR)
monPKD1_flox_Ex4_3_BamHI_BbsI_F, R	GAAGACAAGGATCCCCTTGCTGGACAGTGACTG	GAACAGAAGGACAGGCAGGCGAAG	Long ssODN production (2nd PCR)
