# Supplementary material for: Monkeys mutant for PKD1 recapitulate human autosomal dominant polycystic kidney disease
Source: Nat Commun. 2019 Dec 11;10:5517. doi: 10.1038/s41467-019-13398-6 (PMC6904451; doi:10.1038/s41467-019-13398-6)
Supplement: Supplementary file 3 — Reporting Summary [file 41467_2019_13398_MOESM3_ESM.pdf]

nature research

Corresponding author(s): Masatsugu Ema
Tomoyuki Tsukiyama

Last updated by author(s): Jun 6, 2019

Reporting Summary

Nature Research wishes to improve the reproducibility of the work that we publish. This form provides structure for consistency and transparency in reporting. For further information on Nature Research policies, see Authors & Referees and the Editorial Policy Checklist .

Statistics

For all statistical analyses, confirm that the following items are present in the figure legend, table legend, main text, or Methods section.

n/a	Confirmed	
☐	☒	The exact sample size (*n*) for each experimental group/condition, given as a discrete number and unit of measurement
☐	☒	A statement on whether measurements were taken from distinct samples or whether the same sample was measured repeatedly
☐	☒	The statistical test(s) used AND whether they are one- or two-sided<br>*Only common tests should be described solely by name; describe more complex techniques in the Methods section.*
☒	☐	A description of all covariates tested
☒	☐	A description of any assumptions or corrections, such as tests of normality and adjustment for multiple comparisons
☐	☒	A full description of the statistical parameters including central tendency (e.g. means) or other basic estimates (e.g. regression coefficient) AND variation (e.g. standard deviation) or associated estimates of uncertainty (e.g. confidence intervals)
☐	☒	For null hypothesis testing, the test statistic (e.g. *F*, *t*, *r*) with confidence intervals, effect sizes, degrees of freedom and *P* value noted<br>*Give P values as exact values whenever suitable.*
☒	☐	For Bayesian analysis, information on the choice of priors and Markov chain Monte Carlo settings
☒	☐	For hierarchical and complex designs, identification of the appropriate level for tests and full reporting of outcomes
☒	☐	Estimates of effect sizes (e.g. Cohen's *d*, Pearson's *r*), indicating how they were calculated

Our web collection on statistics for biologists contains articles on many of the points above.

Software and code

Policy information about availability of computer code

Data collection	BD CellQuest Pro for FACS data collection.
Data analysis	FlowJo V9 was used for FACS data analysis.<br>GraphPad Prism 7 was used for statistical analysis.<br>ImageJ was used for image analysis.

For manuscripts utilizing custom algorithms or software that are central to the research but not yet described in published literature, software must be made available to editors/reviewers. We strongly encourage code deposition in a community repository (e.g. GitHub). See the Nature Research guidelines for submitting code & software for further information.

Data

Policy information about availability of data

All manuscripts must include a data availability statement. This statement should provide the following information, where applicable:

- Accession codes, unique identifiers, or web links for publicly available datasets
- A list of figures that have associated raw data
- A description of any restrictions on data availability

The datasets generated or analyzed during the current study are available from the corresponding author on reasonable request.

Field-specific reporting

Please select the one below that is the best fit for your research. If you are not sure, read the appropriate sections before making your selection.

Life sciences study design

All studies must disclose on these points even when the disclosure is negative.

Sample size	The minimum number of animals necessary to achieve the scientific objectives was used because of the ethical reason.
Data exclusions	No data were excluded.
Replication	Some experiments producing the key data were repeated by different co-authors.
Randomization	Multiple animals were used for each biological replicate.
Blinding	All data were checked by multiple individuals who didn't know the genotype of animals.

Reporting for specific materials, systems and methods

We require information from authors about some types of materials, experimental systems and methods used in many studies. Here, indicate whether each material, system or method listed is relevant to your study. If you are not sure if a list item applies to your research, read the appropriate section before selecting a response.

Materials & experimental systems

n/a	Involved in the study	
☐	☒	Antibodies
☒	☐	Eukaryotic cell lines
☒	☐	Palaeontology
☐	☒	Animals and other organisms
☒	☐	Human research participants
☒	☐	Clinical data

Methods

n/a	Involved in the study	
☒	☐	ChIP-seq
☐	☒	Flow cytometry
☒	☐	MRI-based neuroimaging

Antibodies

Antibodies used

Primary antibodies used in this study were as follows:
anti-PC1 (7E12) (sc-130554, 1:10 dilution in IF, 1:100 dilution in WB, Santa Cruz Biotechnology),
anti-PC1 (E8) (8C3C10, 1:3000 dilution, Baltimore PKD Core Center),
anti-PC1 (5F4D2) (MABS1252, 1:200 dilution, Millipore),
anti-Ki67 (M7240, 1:200 dilution, Dako),
anti-AQP1 (ab15080, 1:500 dilution, Abcom),
anti-AQP1 (HPA019206, 1:1000 dilution, SIGMA),
anti-AQP2 (A7310, 1:200 dilution, SIGMA),
Biotinylated LTL (B-1325, 1:300 dilution, Vector),
anti–E-Cad (AF748, 1:100 dilution, R&D),
anti-UMOD (HPA043420, 1:1000 dilution, SIGMA),
anti-NCC (HPA028748, 1:200 dilution, SIGMA),
negative control mouse IgG1 (X0931, 1:5 dilution, Dako),
and negative control rabbit immunoglobulin fraction (X0936, 1:3000 dilution, Dako).

Secondary antibodies used in this study were as follows:
Goat anti-Mouse IgG (H+L) Secondary Antibody, HRP (62-6520, 1:500 dilution, Thermo Fisher Scientific),
Alexa Fluor 488 Donkey Anti-Rabbit IgG(H+L) (A21206, 1:500 dilution, Thermo Fisher Scientific),
Alexa Fluor 488 Donkey Anti-Goat IgG(H+L) (A110055, 1:500 dilution, Thermo Fisher Scientific),
Alexa Fluor 546 Streptoavidin Conjugate (S11225, 1:500 dilution, Thermo Fisher Scientific),
Alexa Fluor 594 Donkey Anti-Mouse IgG(H+L) (A21203, 1:500 dilution, Thermo Fisher Scientific),
Alexa Fluor 633 Donkey Anti-Goat IgG(H+L) (A21082, 1:500 dilution, Thermo Fisher Scientific),
and Alexa Fluor 647 Donkey Anti-Mouse IgG(H+L) (A31571, 1:500 dilution, Thermo Fisher Scientific).

Validation

We performed the staining with negative control IgG antibodies.

Animals and other organisms

Policy information about studies involving animals; ARRIVE guidelines recommended for reporting animal research

Laboratory animals	Cynomolgus monkeys
Wild animals	This study did not involve wild animals.
Field-collected samples	This study did not involve samples collected from the field.
Ethics oversight	All animal experimental procedures were approved by the Animal Care and Use Committee of Shiga University of Medical Science (approval number: 2015-5-13, 2016-6-1).

Note that full information on the approval of the study protocol must also be provided in the manuscript.

Flow Cytometry

Plots

Confirm that:

- [x] The axis labels state the marker and fluorochrome used (e.g. CD4-FITC).
- [x] The axis scales are clearly visible. Include numbers along axes only for bottom left plot of group (a 'group' is an analysis of identical markers).
- [x] All plots are contour plots with outliers or pseudocolor plots.
- [x] A numerical value for number of cells or percentage (with statistics) is provided.

Methodology

Sample preparation	Cells were dissociated and filtered into single cells with trypsin.
Instrument	BD FACS Calibur
Software	BD CellQuest Pro for data collection;<br>FlowJo V9 for data analysis
Cell population abundance	Cell sorting was not performed.
Gating strategy	Doublets and debris were excluded using FSC/FSC-H and dead cells were excluded by propidium iodide. To defined the boundaries, nonstaining samples were used to set the gate.

- [ ] Tick this box to confirm that a figure exemplifying the gating strategy is provided in the Supplementary Information.
